# Towards a Personalized Multi-Domain Digital Neurophenotyping Model for the Detection and Treatment of Mood Trajectories

**DOI:** 10.3390/s20205781

**Published:** 2020-10-12

**Authors:** Yaron Sela, Lorena Santamaria, Yair Amichai-Hamburge, Victoria Leong

**Affiliations:** 1 The Research Center for Internet Psychology (CIP), Sammy Ofer School of Communication, Interdisciplinary Centre (IDC), Herzliya 4610101, Israel; yaron.sela01@post.idc.ac.il (Y.S.); yairah@idc.ac.il (Y.A.-H.); 2 Cubric, School of Psychology, Cardiff University, Cardiff CF24 4HQ, UK; santamariacovarrubiasl@cardiff.ac.uk; 3 Division of Psychology, Nanyang Technological University, Singapore S639818, Singapore; 4 Department of Psychology, University of Cambridge, Cambridge CB2 3EB, UK

**Keywords:** digital phenotyping, mood disorders, neurosensors, dual-EEG

## Abstract

The commercial availability of many real-life smart sensors, wearables, and mobile apps provides a valuable source of information about a wide range of human behavioral, physiological, and social markers that can be used to infer the user’s mental state and mood. However, there are currently no commercial digital products that integrate these psychosocial metrics with the real-time measurement of neural activity. In particular, electroencephalography (EEG) is a well-validated and highly sensitive neuroimaging method that yields robust markers of mood and affective processing, and has been widely used in mental health research for decades. The integration of wearable neuro-sensors into existing multimodal sensor arrays could hold great promise for deep digital neurophenotyping in the detection and personalized treatment of mood disorders. In this paper, we propose a multi-domain digital neurophenotyping model based on the socioecological model of health. The proposed model presents a holistic approach to digital mental health, leveraging recent neuroscientific advances, and could deliver highly personalized diagnoses and treatments. The technological and ethical challenges of this model are discussed.

## 1. Introduction

One of the most prominent characteristics of the current environment is high digital connectivity. This connectivity enables a moment-by-moment quantification of individual-level human phenotypes in situ using data from personal digital devices, both passively and actively. The phrase digital phenotyping has been coined for this process [1]. The rapid growth of embedded smart sensors that are located in wearable technologies and mobile devices allows for the unobtrusive collection of behavioral (e.g., speech patterns), physiological (e.g., heart rate variability), and social activity (e.g., social media use) markers [2,3]. This integrated sensor-based data can be used for the early diagnosis and continuous monitoring of mental health conditions in real-time. Deep digital phenotyping (i.e., in the diagnosis phase) may pave the way for personalized treatment interventions that comprehensively create a full patient journey throughout the care stages (i.e., during the treatment phase). Many of these interventions could be delivered digitally, using one or more of the following techniques: self-help apps, digital cognitive-behavioral therapy, relaxation tools, video-based guidance, virtual-reality, brain–computer interface (BCI) technology, and others [4,5,6]. A better fit between the patient’s unique profile (e.g., sociodemographic factors, clinical characteristics, personality) and treatment enhances the patient’s adherence and therefore maximizes treatmenteffectiveness. Machine learning techniques could contribute to the establishment of a positive feedback circle that enables the constant improvement of treatment interventions for a specific individual [7].

The digital phenotyping approach has been widely applied to the field of mood disorders, such as depression, stress, and anxiety [8,9]. For example, depressive symptoms were found to be correlated with low mobility, as indicated by Global Positioning System (GPS) signals [10], visual cues as indicated by cameras [11], and low social communication patterns as indicated by the frequency of sending messages and phone calls [12]. Most current applications aim to diagnose and deliver treatments in real life, typically using personal devices such as smartphones and wearable devices. However, the current digital phenotyping approach is limited in several ways, as follows.

First, mood fluctuates frequently across time and situations. Hence, reliable real-life diagnoses of mood states must capture within-context and cross-context variability appropriately (e.g., using within and across context (WAC) variability models [13]). The exclusive use of personal devices for digital phenotyping focuses solely on intra-individual variability but neglects the effects of social interactions. Even the social signals that are already obtained (i.e., calls, messages, and social media use) are limited to the communication patterns captured by an individual device, while they disregard other social interactions (e.g., face-to-face conversations). Second, many digital apps for treating mood disorders typically rely on patients to self-report their symptoms. These self-report data are subjective and may be inaccurate due to patient recall bias and compliance issues [14]. Therefore, for accurate diagnosis and treatment, more objective and reliable measures of mental states must be used. For example, according to the electronic ecological momentary assessment approach, continuous, real-time monitoring of various mood biomarkers using sensors and neuroimaging data—particularly electroencephalography (EEG)—can inform a reliable comprehensive clinical diagnosis [15]. Going beyond the clinic, brain–computer interface (BCI) neurotechnology has also been suggested for use in everyday contexts, such as in car emergency braking systems, airplanes, ATM interfaces, and other safety-critical situations [16,17]. Third, the use of smartphones and other wearable devices usually begins in early adolescence. Hence, data from earlier crucial stages of psychological development rely exclusively on self-reports from parents and/or the individual later in life. These significant limitations require a reconsideration of a better digital phenotyping model.

## 2. Moving Beyond the Individual to a Multi-Domain Neurophenotyping Model

**Figure 1:**
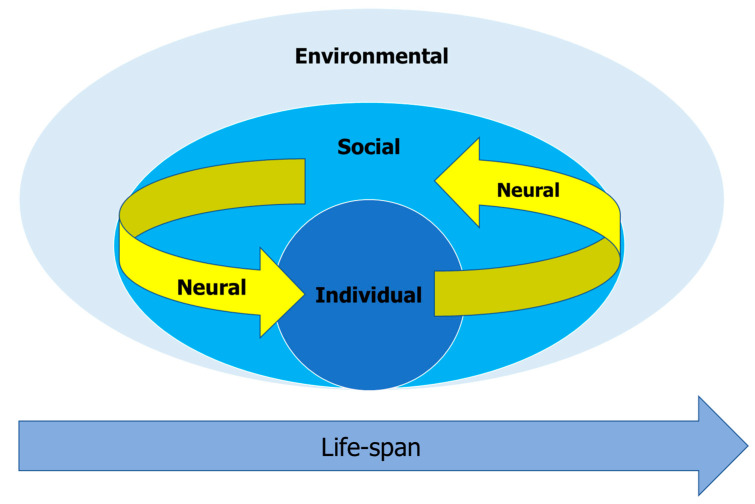
Multi-domain digital neurophenotyping model for sensor-based data collection, analysis, and integration using individual, social, neural, environmental, and life-span domains.

According to the socioecological model of health [18,19], in order to understand patient behavior and implement effective interventions, three levels should be taken into consideration: (1) *Patient-level factors*, which relate to the patient’s attitudes regarding health, in addition to clinical and demographic characteristics; (2) *Micro-level factors*, which relate to interpersonal relationships with health care professionals and social support; (3) *Meso-level factors*, which refer to the characteristics of the health care organization where the patient is being treated; and (4) *Macro-level factors*, which include the characteristics of the health care ecosystem in which a patient lives. Applying and expanding the theoretical framework of the socioecological model, we argue that in order to increase the accuracy of mood trajectory detection, and to improve the personalization of digital treatment, a broader perspective of digital phenotyping must be employed. Thus, we suggest that digital phenotyping should include five complementary domains of data collection and analysis: (1) individual, (2) social, (3) neural, (4) environmental, and (5) life-span domains (see Figure 1):

### 2.1. Individual (Patient)

This level refers to electronic self-reports, behavioral data, and physiological measures gathered from personal devices. These metrics are used not only to detect mood trajectories, but also to understand them in the context of personality traits. Personality is defined as characteristic patterns of thoughts, feelings, and behaviors that a person displays over time and across situations [20]. Personality traits play an important role in predicting mood disorders, as well as the compliance and effectiveness of treatments [21]. Therefore as detected by individual digital footprints, personality traits could serve as a top-down organizing system to improve the personalization of psychological interventions [22,23].

### 2.2. Social

Humans are social creatures, and therefore the interpersonal environment has a major influence on individual affective experience and well-being. Specifically, changes in the frequency and quality of social interactions are strong indicators for mood trajectories [24]. Hence, the inclusion of social indicators in any diagnostic schedule is important in order to establish a broader view of an individual’s affective state. Recent technological and theoretical advancements in social neuroscience have significantly improved our understanding of and ability to measure concurrent social behavior of interacting partners using a sociometric approach [25]. Data from social communication and interaction patterns (e.g., gaze, posture, arousal, speech) may significantly improve the predictive power of digital phenotyping in ecological and naturalistic settings.

### 2.3. Neural

Electroencephalography (EEG) has emerged as a promising objective biomarker for a wide spectrum of psychiatric disorders, including depression [26], bipolar disorder [27], anxiety [28], and obsessive–compulsive disorders [29], among others. The diagnostic potential of EEG signals has already been demonstrated in adults [30], and more recently in children and adolescents [31]. Traditionally, lab-based EEG measurements have relied on the use of simple but well-validated neural markers of mood and affective processing such as frontal alpha power asymmetry [32,33], which is known to index the neural processing of positive/negative emotional stimuli as well as approach/withdrawal behavior. However, recent advances in affective brain computing have increasingly demonstrated the power of deep learning methods to uncover complex patterns in neural activation that can sensitively distinguish between different affective states [34]. This new generation of affective brain–computer interface (BCI) technology can be implemented in wearable EEG systems to monitor emotions in real time while watching a video, listening to music, or experiencing virtual reality, and could be used to inform home-assisting technologies that provide feedback to the user [35,36]. Another potential use of affective BCIs is in emotion regulation, such as the use of music to modulate or enhance one’s mood [37,38]. Some commercially available devices already implement this technology, such as the ‘*Mico LTD*’, a headphone from the company Neurowear that selects music based on the wearer’s mood.

Furthermore, in the fast-advancing field of social neuroscience, dual-EEG (concurrent neural recording from two interacting individuals) is increasingly being used in naturalistic settings to track dynamic changes in interpersonal neural coupling, even between infants and adult caregivers [39,40,41]. Although there are unique technical challenges associated with the collection and interpretation of naturalistic dual-EEG data, particularly with infant participants [42], the mother–infant interpersonal neural network has been found to be exquisitely sensitive to changes in maternal emotional state [43] or parenting stress [44], and also predicts the likelihood of infant social learning from their parent [41]. Dual-EEG studies with adults have demonstrated that interpersonal neural coupling may also index empathy, differentiate the emotional tone of the conversation [45], and signify the degree of cooperation between interacting members of a dyad [46,47]. There is immense potential for these frontier technologies in social and affective neuroscience to be harnessed to enhance the detection and personalized treatment of mood disorders, and to be deployed in closed-loop BCI systems that could function as “emotional prostheses”.

### 2.4. Environmental

Rapid technological progress in the internet of things (IoT), the automobile industry, and in smart cities has created a fabulously rich sensor environment which gathers vast amounts of information that could be associated with mood trajectories. The IoT has especially revolutionized human–computer interactions in indoor settings, by improving sensing and responding to the user. Today, many home products (e.g., refrigerators, home audio systems) include sensitive sensors that measure light, temperature, voice and other bio-physiological metrics. These indicators go far beyond the embedded sensors of personal devices, and therefore provide additional data that indicate the behavioral and emotional states of the user. Initial findings show that dynamically adapting the house environment in response to the individual’s mental state could increase their well-being [48]. In addition, sensors embedded in vehicles constantly measure both active (e.g., reaction time) and passive (e.g., temperature) metrics, which could be linked to the driver’s emotional state [49]. Several studies have already indicated the feasibility of integrating physiological and environmental data to improve the characterization and monitoring of the user’s mental state [50,51,52], or even shape it by delivering in-vehicle interventions [53]. Taking this approach one step further, smart cities could leverage the deployment of a connected network of physical sensors embedded in the environment. This network could provide a novel framework feeding end-users with innovative, smart and efficient services. Further, the large number of behavioral indicators gathered via these sensors (e.g., walking speed, frequency, and quality of social interaction) could support the individual and social domains toward more accurate digital phenotyping.

### 2.5. Life-Span

As mental health is influenced by life events across time, a holistic model of digital phenotyping must include a life-span layer. Applying a developmental approach, a centralized monitoring system that tracks the individual across very long time periods could effectively learn the factors that influence developmental changes in mood trajectories. Specifically, analyzing markers of cognitive, emotional, and social development in the early years could help to more accurately predict the onset of social difficulties and emotional instability in real-life settings [25].

The need for effective mental illness surveillance to improve individual coping, in addition to reducing public burden, has been widely discussed [54,55]. The proposed multi-domain system addresses existing gaps in the detection, monitoring, and treatment of individuals who suffer from mood disorders, and who do not yet receive appropriate mental health support. Specifically, three main populations are expected to significantly benefit from such a system. First, individuals who are not diagnosed with mood disorders due to low accessibility to mental health services or low awareness of the symptoms of mood disorders (estimated at 66% of individuals in the community who suffer from depression [55]). The proposed system could track individual behavioral and physiological signs and prompt the individual when necessary to seek treatment. Second, individuals who suffer from mood disorders and do not receive any treatment (estimated at 35% of adults with a major depressive episode [56]). The system is designed to tailor digital treatments that could be delivered through smartphones and other personal devices, in order to be immediately accessible to these individuals. Third, individuals who were diagnosed and previously received a treatment (pharmacological or psychological) but demonstrated low adherence to the treatment (estimated at 50% of patients receiving antidepressant therapy that discontinue medications in the first four months [57]). Creating personalized treatment interventions could significantly increase patient adherence and thereby improve treatment effectiveness at both individual and public levels.

## 3. Challenges and Considerations

The multi-domain approach that we have proposed will exponentially increase the amount of data collected and analyzed about each individual. This brings with it attendant challenges. With the proliferation of data dimensions and complexity, big data and machine learning analytical methods will become necessary to mine datasets for indicator variables. The big data approach permits the emergence of data-driven discoveries, rather than relying solely on (more limited) theory-driven methods. A strength of this approach is its avoidance of confirmation bias effects; however, there are still important limitations. First, the large amounts of data can lead to the identification of minor effects that only explain a small fraction of variance in the data and are not functionally meaningful. Another problem of high data dimensionality is the presence of confounding factors. For example, variables believed to be independent may not in fact be independent because of the presence of common noise or other factors that generate false associations [58]. To avoid these pitfalls, different sources of data (e.g., neural and behavioral data) can be jointly modeled in order to reduce the number of output variables and increase interpretability.

Furthermore, the use of machine learning for classification of affective states brings particular challenges on top of habitual problems such as the optimization of feature selection to avoid overfitting. The major problem is that the annotation of emotional states in most cases depends on participant self-assessment, which raises issues with validity and bias. Recent studies have proposed different models to improve the emotional labeling of real-life affective situations [59,60] in contrast to single labeling from the valence–arousal space. Other studies have appropriated well-defined classification algorithms from motor imagery and applied these to the identification of emotions, specifically using EEG oscillations (which are not affected by rater bias) to improve feature selection and accuracy [61]. These approaches have included the use of common spatial patterns [62] or Riemannian geometry [63].

The current proposed framework and its eventual deployment in real-life settings relies on the availability of smart, wearable, and comfortable sensor devices—particularly wearable EEG sensors. Although there has been momentum in the so-called “dry revolution” movement toward the use of dry EEG sensors for this purpose [64], the goal of a truly wearable EEG device that delivers high signal quality without compromising user comfort is still some distance from being achieved [65,66]. There are further challenges pertaining to the integration and storage of data. Different sensors may sample information at different timescales and record data in different formats. The integration of information across different sensors will require a data architecture that synchronizes and aligns the various inputs and allows the sensors to work in combination. In addition, the enormous amounts of data that must be stored and managed will require smart storage solutions.

Alongside the technological barriers, three important ethical concerns need to be addressed. First, the process of reliable digital phenotyping requires that at least part of the gathered data must be personally identifiable. Second, the collection and handling of such personally identifiable and sensitive data presents the possibility for abuse and exploitation, by parties that may include commercial companies and governmental agencies. Finally, despite efforts to implement learning via a neutral artificial intelligence that would be guided by ethical considerations, social inequalities and exclusions may inadvertently be perpetuated due to human biases embedded in the algorithms. The serious ethical concerns of digital health systems have been highlighted before [67]. Among these solutions, we suggest employing robust encryption protocols throughout sensors, data storage, and management systems, in addition to building a decentralized system architecture that might mitigate the risk of information exploitation.

Despite these significant challenges, a multi-domain digital neurophenotyping model holds great promise for the future of mental health, particularly for the diagnosis of mood disorders and promotion of mental well-being. Applying a holistic approach and integrating the available data sources across domains could contribute to early and more accurate diagnostic procedures and provide personalized and therefore more effective treatment interventions.
